# Interpopulation Variation in Egg Mortality in Response to Hot and Dry Conditions in North American Populations of the Invasive Tiger Mosquito, *Aedes albopictus*

**DOI:** 10.1002/ece3.73557

**Published:** 2026-04-23

**Authors:** Katie M. Westby, Laura Tayon, Benjamin Orlinick, Angela Smith, Kim A. Medley

**Affiliations:** ^1^ Tyson Research Center Washington University in Saint Louis Eureka Missouri USA; ^2^ Medical Scientist Training Program Emory University School of Medicine Atlanta Georgia USA; ^3^ St. Tammany Parish Mosquito Abatement District Slidell Louisiana USA

**Keywords:** *Aedes*, carry‐over effects, common garden, insect egg desiccation, relative humidity, temperature, thermal tolerance

## Abstract

How wild organisms are responding to climate change is one of the most pressing issues of the 21st century. As ectotherms, insects are particularly vulnerable to increases in temperature and extreme weather events because their fitness and survival are directly linked to abiotic conditions. For holometabolous insects with a wide geographic range, it is important to quantify upper thermal and lower hydric limits for multiple populations and life stages that differ in habitat usage and mobility. Population differences in egg survival are understudied despite the importance this life stage plays in population dynamics. As a sessile life stage, eggs likely experience strong selection pressures in response to extreme heat and drought. In this paper, we tested for population and phenotypic differences in egg survival for multiple USA populations of the invasive mosquito vector, *Aedes albopictus* . We found evidence of interpopulation differences in egg survival in response to high temperature and low humidity as well as plasticity in survival depending on experimental conditions. Some populations consistently had low survivorship across experiments. We found that field‐collected eggs have low survivorship under field conditions, suggesting that egg survivorship may be low in nature. Finally, we showed that eggs experiencing high temperatures produced larvae that were unable to develop to the fourth instar during a simulated heatwave. Combined, our results reveal phenotypic plasticity and suggest a heritable component to thermal and hydric tolerance in this globally invasive species.

## Graphical abstract

North American populations of the invasive tiger mosquito exhibit genetic and phenotypic differences in egg survival in response to hot and dry conditions.

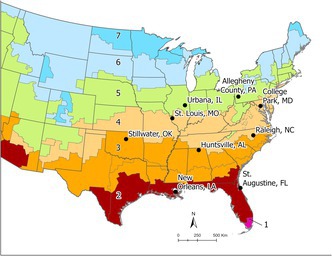

## 1 Introduction

The rapidly changing climate is one of the most important drivers of human‐induced global change. Increases in average temperatures, coupled with increased frequencies of extreme events such as droughts, floods, heatwaves, and destructive storms, have put additional pressure on species and populations already grappling with habitat loss, pollution, and invasive species. Empirical examples of reductions in populations and species in response to extreme climatic events are mounting (Buckley and Huey 2016; Maxwell et al. 2019; Ma et al. 2021), and while we may currently lack universal definitions of what extreme events are (Bailey and van de Pol 2016), there is an urgent need to quantify both the short and long‐term consequences on individuals, populations, and communities.

Insects, as largely ectothermic organisms, are particularly vulnerable to climatic extremes, and large‐scale declines in insect biomass and diversity have been documented (Wagner 2020; Harvey et al. 2023). Many insect taxa have complex life histories with immature and adult stages occupying different niches, often with stages that are mobile (e.g., adults) and sessile (e.g., eggs). This will govern which life stages have the capacity to behaviorally thermoregulate by moving to more favorable microclimates during times of environmental stress. Thus, in addition to species and populations experiencing different selection pressures in response to climate change, individual holometabolous insects will experience climate differently across life stages, with consequences for local adaptation. Additionally, the extent to which individuals will be able to behaviorally adapt to climate change, such as changing the daily or seasonal timing of behaviors (Abbasi 2025), will also be life stage dependent. Thus, to accurately predict how a species will respond to climate change, it is necessary to quantify thermal and hydric limits across multiple populations and life stages (Kingsolver et al. 2011; Radchuk et al. 2013; MacLean et al. 2016; Austin and Moehring 2019) and potential carry‐over effects on subsequent life stages.

In recent years, more research has been conducted to assess the upper thermal limits of many insect taxa and to test for evidence of local adaptation. Most of these studies focus on adult and larval stages in laboratory assays, but considerably less is known about egg survival in response to hot and dry conditions (Klockmann and Fischer 2017). Eggs, which for essentially all insects are immobile and thus lack the ability to behaviorally thermoregulate, rely on maternal partitioning of resources such as heat shock proteins, lipids, and hydrocarbon coatings of the shell (Urbanski et al. 2010; Farnesi et al. 2015) as well as other maternal egg‐laying strategies that maximize survival (Hilker et al. 2023; Li et al. 2025). For example, females often choose to oviposit in habitats without predators or with fewer conspecific competitors; however, empirical evidence on how abiotic conditions affect oviposition choices in insects is lacking (Hilker et al. 2023, Li et al. 2025).

Because of differences in selection pressures by life stage, adults and eggs of the same population may respond differently to the climatic conditions they experience (Borda et al. 2021). Heat stress, especially when coupled with dry conditions, has been shown to be lethal to eggs in a variety of taxa, including ticks (Ajayi et al. 2024) and mosquitoes (Sota and Mogi 1992a; Juliano et al. 2002; Faull and Williams 2015; Faull et al. 2016) and can have downstream effects on the next life stage (Potter et al. 2011; Klockmann et al. 2017). Importantly, heat stress experienced during the egg stage may interact with other pressures with detrimental consequences for later stages (Janssens et al. 2017). However, much of what we know regarding egg survival and local adaptation concerns the diapause trait in response to winter. Latitudinal clines in the diapause response are well documented and allow for greater winter survival in cold climates (Urbanski et al. 2012; Medley et al. 2019). A handful of recent studies have found evidence of population differentiation in upper thermal tolerance in *Drosophila* (Lockwood et al. 2018; Borda et al. 2021) and mosquito eggs (Reidenbach et al. 2014; Faull et al. 2016), although this response is not consistent for all insect taxa (e.g., *Manduca sexta* eggs, Potter and Woods 2012).

The consequences of egg mortality for population and community dynamics are also understudied. Though it can be inferred that egg survival is an important component of population growth, this life stage is often overlooked, or mortality is assumed to be constant in stage‐structured matrix models (but see Radchuk et al. 2013). Additionally, the effects of egg mortality on community interactions are largely unexplored, likely because eggs are not actively consuming resources in the environment. However, reductions in egg survival may change the strength of interactions among feeding larvae, and there is some empirical evidence that it can reverse the outcome of interspecific interactions in communities (Costanzo et al. 2005). For example, differential egg mortality in response to drought for two species that compete as larvae in aquatic habitats reversed the competitive outcome for the mosquito *Aedes albopictus* and allowed for coexistence with the ecologically similar mosquito *Aedes aegypti* (Juliano et al. 2002; Costanzo et al. 2005). Here, we explore egg mortality of multiple populations of the invasive tiger mosquito, *Aedes albopictus* , to test for population‐level variation in responses to heat stress and low humidity under a range of environmental conditions. Studying population differences in egg mortality is necessary for predicting future range expansions and contractions, which takes on special urgency when considering pest and vector species.

In this paper, we tested for differences in egg mortality in response to low humidity and moderate to high temperatures from nine populations across *Ae. albopictus*' US range. Our previous work demonstrated that adults of some of these same populations differed in average critical thermal maxima (CTmax) by up to two degrees Celsius but larval populations were more similar, suggesting life stage differences in local adaptation (Orlinick et al. 2024). We build upon that previous work by quantifying egg survival under a range of temperature and humidity conditions in two common garden experiments: one in the laboratory and one in the field. Because eggs were produced under standardized common garden conditions, consistent differences among populations are expected to reflect underlying genetic differences and/or persistent maternal effects. We predicted that, when presented with extreme temperature and humidity conditions, we would see population divergence in egg survival. We also compared survival differences of wild‐collected eggs from three urban populations from one city to our colony strains under field and standard lab conditions. We predicted that wild‐collected eggs would have lower survival than lab colonies, especially under field conditions, and that field conditions would reveal stronger population differentiation than lab conditions because conditions experienced in the field are suboptimal. Finally, in a third experiment, we tested for carry‐over effects of temperature experienced during the egg stage on larval survival during a simulated heatwave. We predicted that high‐temperature egg environments would either (a) prime larvae for improved survival under a heatwave or (b) reduce survival because of accumulated heat damage. This work is important for understanding how this globally important vector and pest species is responding, and will respond, to increases in summer heat, more frequent droughts, heatwaves, and for assessing its adaptive potential if heritable variation is present.

## 2 Methods

### 2.1 Study Species

*Aedes albopictus* is native to Southeast Asia and has spread widely across the globe since the 1980s, including the United States. Recognizable by its white markings on the legs and a white stripe down the center of its thorax, *Ae. albopictus* is an aggressive daytime biter, feeds on a wide variety of hosts, and is a considerable nuisance species (Paupy et al. 2009). Importantly, this mosquito is a known vector of multiple viruses of human health concern, such as dengue, Zika, and chikungunya. In the United States, *Ae. albopictus* can be found in a variety of habitat types but is especially common in urban areas where it experiences the urban heat island effect (Westby et al. 2021). Consequently, its invasion and ecological niche have been well studied (Bonizzoni et al. 2013). One of the keys to its success and global spread is that it lays desiccation‐resistant eggs above the water line in containers, especially in human refuse, such as used tires and buckets, which can survive for months under ideal conditions (Lounibos 2002; Swan et al. 2022). The outer eggshell, or chorion, contains specialized proteins and lipids that reduce water loss, and eggs hatch when inundated with water (Urbanski et al. 2010; Farnesi et al. 2017; Li et al. 2025). However, laboratory survival assays have shown that *Ae. albopictus* eggs are susceptible to death and desiccation, particularly in response to low humidity (Juliano et al. 2002; Martín et al. 2025) but also high temperatures.

### 2.2 Field Collection and Colony Maintenance

**FIGURE 1:**
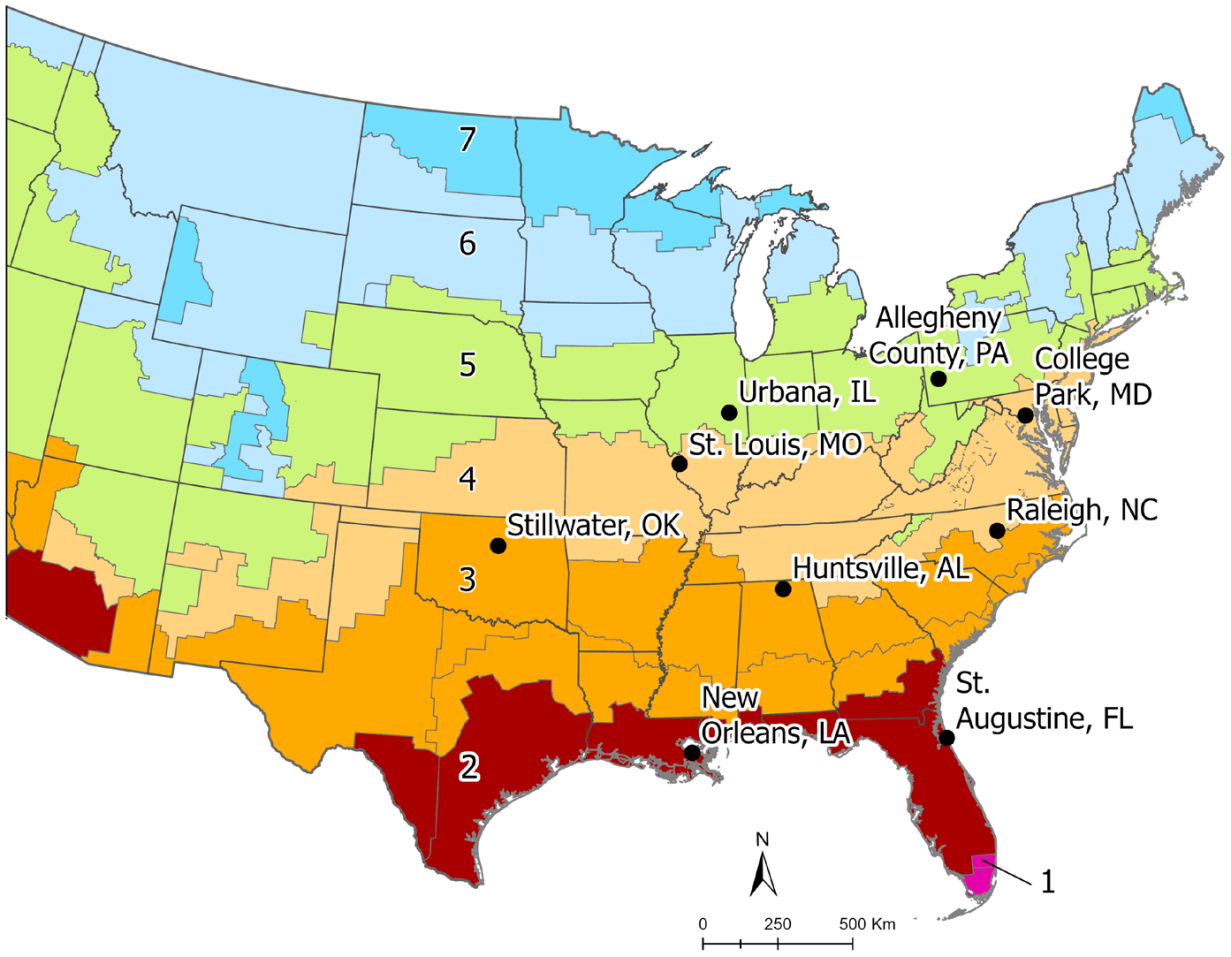
Climate zone map with the populations.

*Aedes albopictus* eggs were collected by colleagues from nine locations across the eastern United States and shipped to Tyson Research Center in Saint Louis, MO, USA. The locations were chosen because they spanned four climate zones across *Ae. albopictus*' eastern range. The climate zones are defined by the International Energy Conservation code and are derived from temperature, humidity, and rainfall patterns (https://codes.iccsafe.org/content/IECC2012P5/chapter‐3‐ce‐general‐requirements) (Table 1, Figure 1).

**TABLE 1:** Climate and geographical information on the nine populations used in experiments.

Climate zone	Climate type	Sample location	Latitude	Longitude
5	Cool‐humid	Allegheny County, PA	40.48 N	80 W
5	Cool‐humid	Urbana, IL	40.11 N	88.2 W
4	Mixed‐humid	College Park, MD	39 N	76.9 W
4	Warm‐humid	St. Louis, MO	38.62 N	90.2 W
3	Mixed‐humid	Stillwater, OK	36.12 N	97 W
3	Warm‐humid	Raleigh, NC	35.78 N	78.64 W
3	Warm‐humid	Huntsville, AL	34.73 N	86.56 W
2	Hot‐humid	New Orleans, LA	29.95 N	90.08 W
2	Hot‐humid	St. Augustine, FL	29.89 N	81.31 W

*Note:* Populations are ordered north to south and spanned much of the Eastern USA range of *Ae. albopictus*. The climate zones and climate types are defined by the International Energy Conservation code.

Mosquitoes were collected in the summer and fall of 2021 using 0.5 L black plastic oviposition cups lined with seed germination paper. Within each location, eggs were collected from multiple sites, from multiple weeks, or both to ensure we captured as much genetic diversity as was present in the population. A minimum of 200 individuals from each site were used to establish each colony. All field (F_0_) collected eggs were hatched in 0.3 g/L Difco nutrient broth with the resulting larvae reared in deionized water (DI) in cups in the lab with *ad libitum* bovine liver powder (MP Biomedicals). Pupae were transferred to cups containing DI water in 30 × 30 cm wire mesh cages, and allowed to eclose freely. One cage was used per population. Once all pupae had eclosed, adults were fed daily on defibrinated bovine blood tied into a ball in hog casing and warmed to 60°C. The blood ball was placed on top of the cage for feeding. Mosquito fecundity is influenced by vertebrate host source (de Swart et al. 2023), so to minimize variation, all females were fed on bovine blood from the same distributor (HemoStat Laboratories, Dixon, CA). It is possible that some populations were intrinsically more or less fecund or produced lower‐quality eggs on bovine blood than others. However, differences in egg quantity and quality by host species have been shown to exist at the level of mosquito species, rather than among populations, and is an area for future research. Additionally, it is common when establishing new colonies that a proportion of females will not mate, producing inviable eggs, or feed effectively on an artificial feeding system. We did not observe any obvious differences, however, between the populations during the early colony establishment phase and only used eggs from generations F_2_–F_4_ for experiments to allow for populations to stabilize and reduce maternal effects. Adults were given constant access to 10% sucrose solution delivered via cotton wicks. Black plastic cups filled with oak leaf infusion and lined with seed germination papers (hereon referred to as egg papers) were kept in the cages to allow for oviposition and were replaced with new papers every 4 days. Removed egg papers were allowed to dry and stored in clear plastic boxes kept at 95%–100% relative humidity. All eggs, larvae, and adults were kept in environmental chambers under standard conditions (16:8 light:dark photophase and 25°C). F_1_–F_3_ generations were reared under the described conditions except that larvae were reared under a standardized feeding protocol to ensure that the eggs used in experiments came from the same nutritional starting point to reduce variation due to maternal effects for our common garden experiments. Standard protocol: when larvae were ≤ 24 h old, they were sieved from the nutrient broth solution and 400 1st instar larvae were counted and placed in clear plastic rearing pans with 3 L DI water and 0.6 g liver powder. On day 9, we added 0.3 g liver powder and fresh DI water to bring the volumes back to 3 L. We did not quantitatively monitor blood feeding rates, fecundity, or survival during the colony establishment or maintenance phase.

### 2.3 Egg Mortality Experiment 1: Lab Survival Experiment

In the laboratory, we set up common gardens with four humidity and two temperature treatments for all nine populations in a factorial design. We created four relative humidity (RH) treatments inside plastic desiccators using saturated salt solutions and DI water in the bottom of the desiccator, separated from the eggs via a perforated divider. The treatments were RH 25% (Lithium chloride), 40% (Potassium formate), 60% (Potassium carbonate), and ~100% (DI water). At ~100% RH, water condensation on egg surfaces can sometimes trigger premature hatching in *Aedes*. While we did not see evidence of this happening in our experiment, we cannot completely exclude the possibility that a small number of eggs hatched before the end of the trial. The salts chosen were based on data reported in Winston and Bates (1960), but in practice, it took several rounds of trial and error to achieve stable RH values within our desired ranges under the various temperature regimes. One desiccator per humidity treatment was placed in each of two environmental chambers set to a 16:8 L:D cycle for the two constant temperature treatments: 28°C and 31°C. The egg papers for each treatment were in a single desiccator and randomly mixed to control for any micro differences in the desiccator. The two constant temperatures were chosen to approximate moderate and relatively high summer temperatures commonly experienced by *Ae. albopictus* in the eastern USA, while remaining within the range of conditions observed in shaded microhabitats. Daily mean and maximum temperatures in our study region during summer often reach 28°C–32°C (Westby et al. 2021, K. M. Westby, unpublished data). Temperature and humidity were monitored with a HOBO data logger (MX2301A) inside each desiccator.

The F_3_ eggs for the experiments came from egg papers collected from the nine colonies (populations) on three different occasions (events) for a total of 27 egg papers. To assign eggs to each treatment group, each egg paper was cut into eight strips that were randomly assigned to each of the treatment groups (4 RH × 2 Temps = 8 treatment groups). This totaled 216 strips (3 events × 8 treatment groups × 9 populations = 216). We took care to ensure that each treatment group received one egg strip from each of the three event papers to distribute the variation in egg age equally among groups and thus egg age was not included in the statistical model because of this balance. The eggs were between 4 and 6 weeks old when the experiment began. Each egg batch was carefully examined under a dissecting microscope and the number of viable eggs were counted (range 6–162). An egg was considered viable if it was plump with no evidence of shriveling or collapsing. Any flat or concave egg on the paper was excluded from the total viable egg count at the start of the experiment. We used this approach because more reliable approaches include either hatching the eggs or bleaching them to determine viability, which renders them unavailable to experiments. We attempted to remove the eggs deemed inviable in a pilot experiment but found that because the eggs on our papers were laid closely together, we also removed many viable eggs and consequently opted for our visualization protocol. To validate our method of visually determining egg viability, we conducted a second pilot run of the visualization protocol and hatched the eggs immediately thereafter to determine proportion of viable eggs to compare to our visual estimates. We used four separate egg papers in our trial and found that our method produced an error rate of 0%–8% difference between the number of viable eggs we counted and the number that hatched (egg totals: % difference; 128: 8%, 27: 0%, 131: 5%, 145: 7%). Only two researchers were responsible for determining and counting the viable eggs for each experiment, so the error should have been distributed equally among treatment groups and therefore not bias our results. Due to the error rate in our methods, there were seven observations with a survival rate above 100%. We incorporated an adjustment to our visual eggs counts by applying a linear model, created from the calculated error rate from our pilot, using the following formula: true egg count = −0.251 + 1.2906 × visual egg count. The regression had an *R* ^2^ of 0.88. The corrected counts were rounded to integers for analysis. Despite the negative intercept, none of the adjusted values fell below zero. This same correction was applied to the egg counts in both experiments one and two as the same researchers performed the egg counts and we assumed that the error rate would be similar. We did not, however, systematically quantify the false‐negative rate for eggs scored as inviable across all populations, so we cannot entirely exclude the possibility that a small fraction of morphologically collapsed eggs remained viable, particularly under certain stressful treatments or that our implicit assumption that the relationship was consistent across treatments is false.

The survival assay was concluded after 21 days, at which point the eggs were stimulated to hatch by submerging them in nutrient broth solution for 24 h. We counted all hatched larvae, re‐dried the paper, and hatched again every 2–4 days until no more survivors hatched.

We analyzed the proportion of eggs that survived using a GLM with a binomial distribution, using RH, temperature, population, and all pairwise interactions as fixed effects. We did not calculate pairwise comparisons among populations within the interactions because we were not specifically interested in which populations were different from one another. Additionally, the number of possible test comparisons within each interaction was over 150, increasing the risk of reduced statistical power and overinterpretation of marginal differences after the multiple comparison correction. All statistical analyses were performed using the GLIMMIX procedure in SAS 9.4.

### 2.4 Egg Mortality Experiment 2: Field Survival Experiment

In this experiment, we assessed egg survival in a common garden in the field using a lab common garden as our control. We used F_4_ eggs from eight of our lab‐reared populations used in experiment 1 and F_0_ eggs collected from three separate locations from the greater St. Louis, MO USA region which served as our field populations (11 populations total). In this way, we were able to test whether wild eggs collected from different urban populations, in the field, survived better or worse than lab colony eggs as well as test those lab populations under realistic conditions. Field‐collected eggs came from three backyards all greater than 5 km apart. Two 6 L black buckets with 4 L of a very diluted hay infusion as an attractant were placed at each site with two pieces of seed germination paper taped in the water line for adult *Ae. albopictus* to lay their eggs. Egg papers were collected three times from each site between June 27 and July 6, 2022 and returned to the lab, where they were stored in a plastic box with 100% RH in a chamber set at 25°C, 16:8 light:dark until the experiment began. For the eight lab‐reared populations, we used the same standardized rearing protocol described above. Each egg paper was carefully inspected before the experiment, and only plump eggs were counted as viable with flat and indented eggs excluded from the total count (see more details about this in the methods for experiment one). Each paper contained 30–100 viable eggs. For the three field‐collected populations, eggs from two collection dates were divided equally between the lab and field treatments so that each population contributed four or more egg papers, each with no fewer than 45 viable eggs. The field treatment took place on a ridge, under the canopy of oak‐hickory forest at Tyson Research Center in Eureka, MO. Egg papers were placed in a large plastic tub with a mesh lid to prevent predation. The tubs were protected from precipitation by an A‐frame tent with clear plastic sheeting open at the sides to allow air flow. Temperature and humidity were recorded every 30 min with a HOBO data logger (MX2301A) inside the tub with the eggs. Lab‐treatment eggs were kept at 25°C and 95%–100% RH in a desiccator in an environmental chamber and served as a control. The survival experiment lasted for 2 weeks (July 13–27, 2022) after which the field eggs were returned to the lab. Our field experiment was 1 week shorter than the 3 weeks we ran our lab experiment because we were concerned that a major flooding event forecast for Saint Louis would be too severe for our plastic canopy rain guard to withstand and so we ended the experiment early. All egg papers (field and lab controls) were submerged in 0.03 g/L Difco nutrient broth solution for ≤ 24 h and the number of hatched larvae were counted. Regular hatching continued until no more eggs hatched.

We analyzed the proportion of eggs that survived, using the same correction as in experiment one, with a GLM with a binomial distribution, with main effects of population, treatment (field or lab), and their interaction. The population term included both the three urban populations and the eight lab populations.

### 2.5 Larval Mortality Experiment: Carry‐Over Effects of Egg Environment on Larval Survival in an Experimental Heat Wave

We tested the effects of conditions experienced by eggs on larval survival during a simulated heatwave on our nine laboratory colonies. We exposed eggs from each colony to two treatment conditions and the subsequent larvae were exposed to one heatwave. For this experiment, a single egg paper with F_2_ eggs from each colony was placed in desiccators set at 60% RH maintained using potassium chloride saturated salt solution. To approximate slightly more realistic temperature conditions, we had different diel and nocturnal temperatures which were based on temperature data collected at field sites in Saint Louis, MO during July 2018 (Westby et al. 2021, K. M. Westby, unpublished data). The egg treatments approximate relatively warm and hot but realistic daily temperature cycles in shaded container habitats. Our treatments had a higher temperature during the 16‐h light cycle in our environmental chambers and a lower temperature during the 8‐h dark cycle. The two treatments were high: 33°C:24°C (mean of 30°C), and low: 29°C:20°C (mean of 26°C). We also had a control treatment which consisted of eggs that were kept in our standard egg storage conditions of 95%–100% RH and a constant 25°C. The egg treatment ran for 21 days, at which time they were hatched in a 0.3 g/L nutrient broth solution. After 24 h, individual larvae were placed in 30 mL vials with 25 mL of 0.2 g/L nutrient broth solution as an artificial food source. A preliminary trial showed that under our standard 25°C control conditions, 85% (*n* = 14) of the larvae survived to adulthood with this food source. Individual larvae were treated as replicates with 9–10 replicates per population and treatment. For four of the populations (College Park, Huntsville, Stillwater, and Urbana) no eggs survived the high temperature egg pretreatment which led to a reduction in the total number of larvae included in the heatwave experiment from a possible 270 larvae (9 populations × 3 egg treatments × 10 replicates = 270 larvae) to 228. The 24‐h old larvae were then placed in our heatwave treatment in a chamber set at 38°C:31°C on a 16:8 light cycle (mean 36°C). This temperature regime was selected to simulate stressful heatwave conditions that neared the upper limits that larvae fail to thrive based on laboratory estimates using constant temperatures (Delatte et al. 2009). Larval mortality was assessed regularly.

No larvae survived to adulthood, so we analyzed the proportion of larvae that survived to the fourth instar using a GLM with a binomial distribution and population and temperature treatment as fixed effects. We did not include the interaction as not every population had surviving larvae from the high temperature egg pretreatment to be included in the larval survival experiment. In total, 228 larvae were included in the experiment included in the survival assay.

## 3 Results

### 3.1 Egg Mortality Experiment 1: Lab Survival Experiment

No eggs survived from the two lowest RH treatments. Because survival was uniformly zero across populations at 25% and 40% RH, these treatments provided no variation for the binomial GLM and were excluded from that analysis. This effectively reduced the size of our experiment to a 2 temperature × 2 RH × 9 population factorial experiment and indicates that continuously holding eggs for 21 days at these low humidities, and the moderate to high constant temperatures used here, is lethal for *Ae. albopictus*.

**FIGURE 2:**
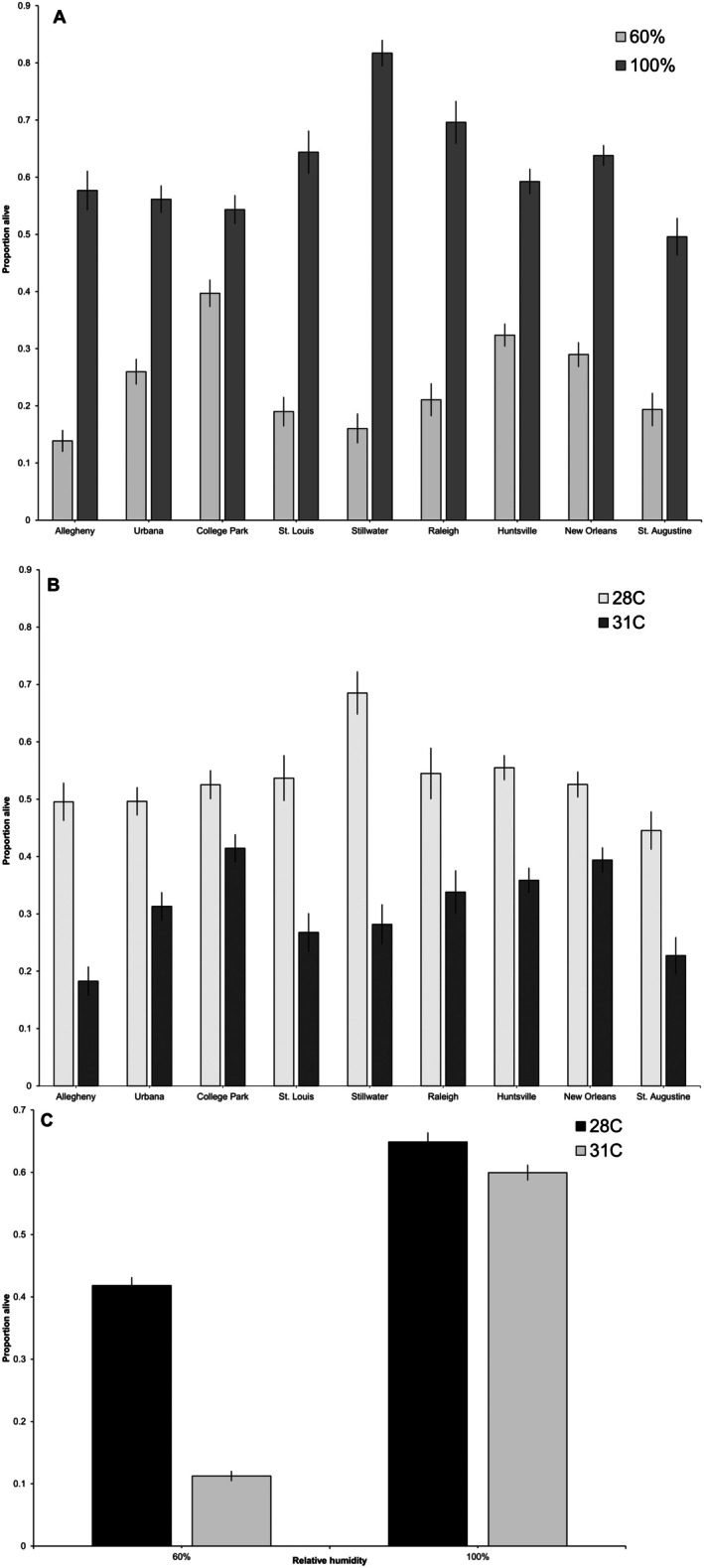
Least squares means and standard errors from the lab experiment. (A) The interaction between population and RH. (B) The interaction between population and temperature. (C) The interaction between temperature and humidity.

The main effects of RH (*F* = 626.94; df 1, 71; *p* < 0.0001), temperature (*F* = 204.19; df 1, 71; *p* < 0.0001), and population (*F* = 6.98; df 8, 71; *p* < 0.0001) were all highly significant as were the interactions: RH*population (*F* = 13.98; df 8, 71; *p* < 0.0001; Figure 2A), temperature*population (*F* = 4.49; df 8, 71; *p* = 0.0002; Figure 2B), and RH*temperature (*F* = 165.79; df 1, 71; *p* < 0.0001; Figure 2C). There was less population differentiation when the RH was high or the temperature moderate (100% RH, Figure 2A and 28°C, Figure 2B), but when conditions were more extreme (60% RH, Figure 2A and 31°C, Figure 2B), we observed population divergence in egg survival. The interaction between RH and temperature revealed that the lowest egg survival (mean 14.4% ± 1%, Figure 2C) occurred when humidity was lower and the temperature was higher. When the RH was ~100%, there was high survival for both temperature treatments which did not differ (76%–77% ± 1%, Figure 2C).

### 3.2 Egg Mortality Experiment 2: Field Survival Experiment

**FIGURE 3:**
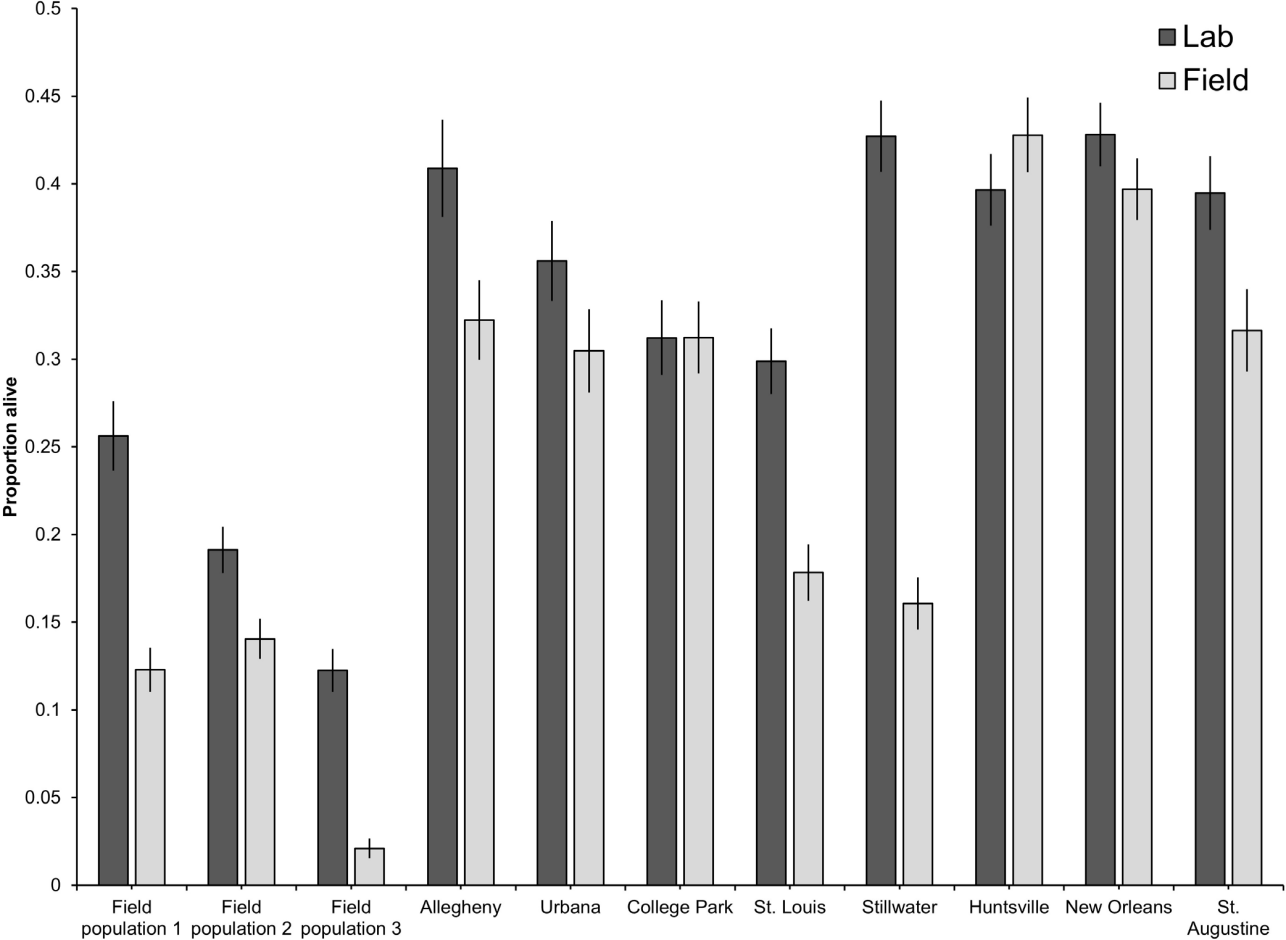
Least squares means and standard errors from the field experiment. “Field” and “Lab” in the figure legend refers to where the treatment took place, not the origins of the eggs. Field populations 1–3 were eggs collected directly from the field from wild mosquitoes and the other populations were reared in the lab using our standard rearing procedure.

**FIGURE 4:**
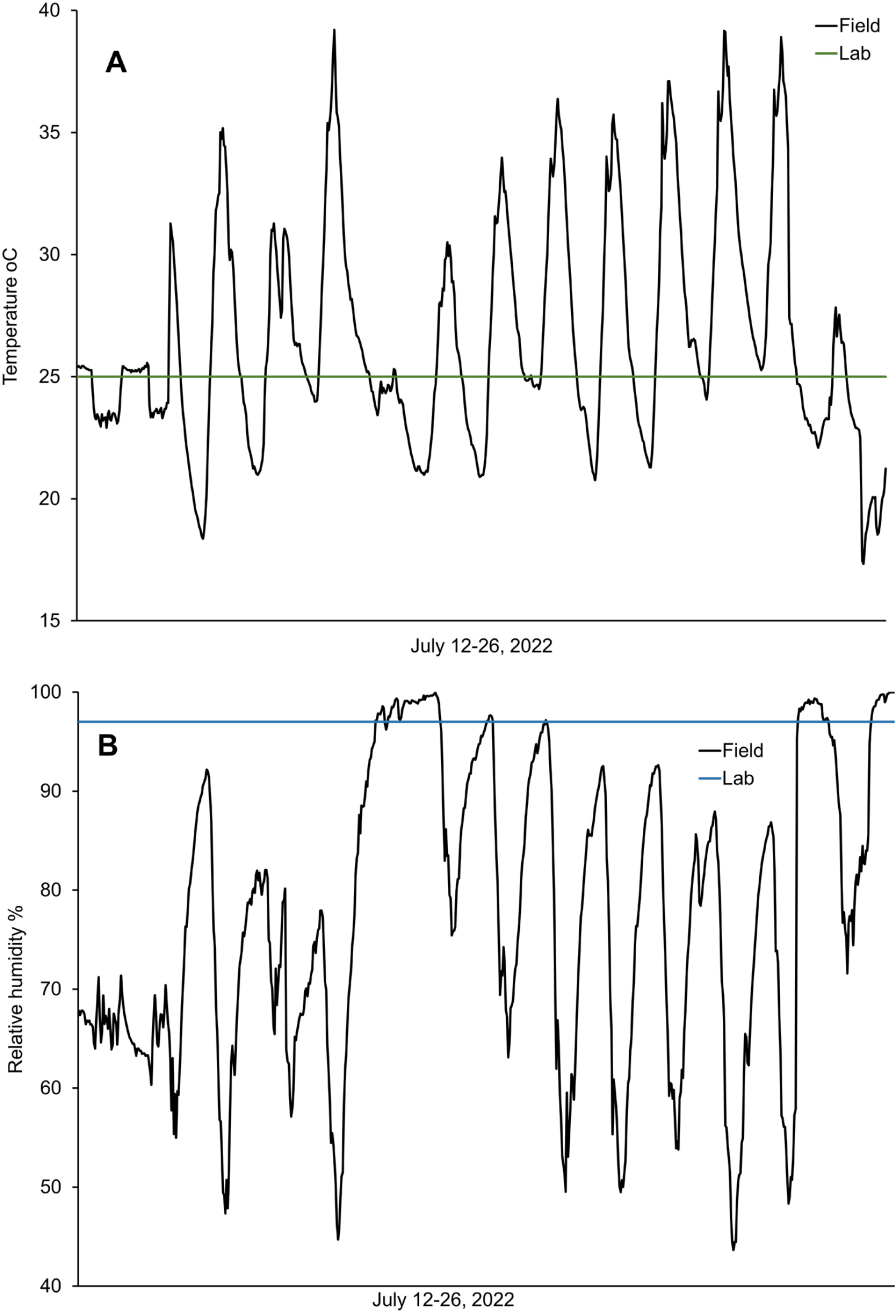
(A) Temperature and (B) RH humidity data recorded directly next to the eggs during our 2‐week field survival experiment.

The effects of population (*F* = 63.09; df 10, 90; *p* < 0.0001), treatment (*F* = 135.70; df 1, 90; *p* < 0.0001), and their interaction (*F* = 12.32; df 10, 90; *p* < 0.0001) were all highly significant (Figure 3). Eggs of all three of the urban populations and three of the lab populations (Allegheny, Saint Louis, and Stillwater) had comparably lower survival in the field compared to the eggs kept under standard conditions in the lab (Figure 3). Eggs of two of the three urban populations had similar survival to all the lab populations except for Stillwater, which showed higher survival (Figure 3). The third urban population had low survival in the lab treatment and almost no eggs survived the field treatment (Figure 3). The temperatures and humidity the eggs experienced during the field survival treatment are shown in Figure 4A,B.

### 3.3 Larval Mortality Experiment Carry‐Over Effects of Egg Environment on Larval Survival in an Experimental Heat Wave

**FIGURE 5:**
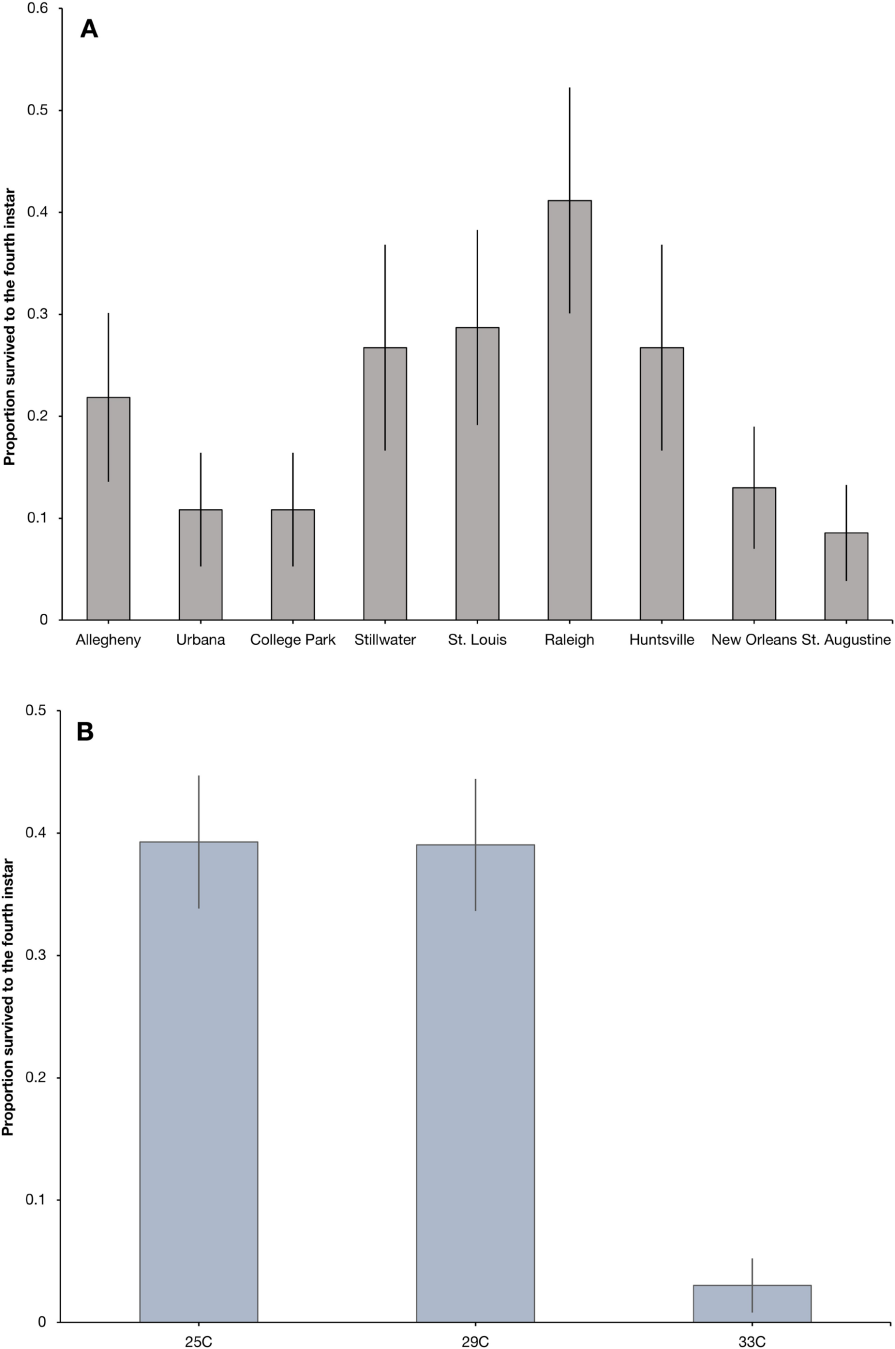
Least squares means and standard errors of the proportion of larvae that survived to the fourth instar in our lab based simulated heatwave experiment. (A) There were no significant differences among populations. (B) The temperature the eggs experienced had a significant effect on the proportion of resulting larvae that survived to the fourth instar in our simulated heatwave.

No larvae survived to adulthood and only 5 larvae pupated, so we analyzed the proportion that survived to the fourth instar. The effect of population on larval survival was not significant (*F* = 2.01; df 8, 12; *p* = 0.133; Figure 5A), but the effect of egg treatment was (*F* = 7.76; df 2, 12; *p* = 0.0069). The low temperature egg treatment and the control treatment had equivalent survival of approximately 40%, but the high temperature treatment had very low survival of approximately 3% (Figure 5B).

## 4 Discussion

The results of our common garden experiments are consistent with population differences in egg mortality that include a heritable component, as well as phenotypic variation, among some North American populations of the tiger mosquito. This finding leads us to hypothesize that there is standing variation in thermal and desiccation tolerance. How species will respond, and are responding, to climate change is one of the most important questions in global change research today. To answer this question, we need a better understanding of the capacity of current populations to respond to warmer temperatures (Rodrigues and Beldade 2020) but also a baseline understanding of whether populations within species have heritable differences in upper thermal tolerance (Diamond 2017), which will be crucial for further adaptation.

For our nine laboratory strains, differences in egg survival among populations were more apparent under stressful conditions (e.g., high temperatures or low humidity). Many studies from diverse taxa find limited differences in upper thermal limits within populations across large geographic areas and have concluded that while population differences along climatic clines may be common for cold tolerance, upper thermal limits are more constrained (Addo‐Bediako et al. 2000; Sunday et al. 2011; Hoffmann et al. 2013). However, any differences, if they exist, may be less apparent for mobile life stages, such as flying adults, which can behaviorally thermoregulate and use cooler microclimate refugia thus reducing the strength of selection (Lockwood et al. 2018). In fact, thermal adaptation can be decoupled in mobile versus sessile life stages in the same species with stronger evidence of thermal adaptation in immobile eggs (MacLean et al. 2016; Borda et al. 2021; Chakraborty et al. 2025). Our own previous work, however, suggests that adult *Ae. albopictus* from the same populations tested in this paper do vary in CTmax but, as with egg survival, there was no clear latitudinal cline in survival (Figure 2A,B; Orlinick et al. 2024). In Orlinick et al. (2024), we were able to show that for adults, temperature in the home climate was not strongly correlated with CTmax. Instead, average annual rainfall and relative humidity were positively correlated with CTmax. We hypothesize that a wetter, more humid climate may ameliorate some of the damaging effects of high temperatures, potentially allowing populations to adapt to withstand them. While this is conjecture and the data presented here and in Orlinick et al. (2024) are inadequate to test the idea, it does highlight the importance of precipitation and relative humidity in insect survival. Unfortunately, we were unable to run models of egg survival with the same climate variables as we did with adults due to the more complicated experimental design used here.

Interestingly, when we ignored variation in egg survival by population and looked at the interaction between temperature and humidity, we found that high humidity mitigated the negative impact of a 3°C increase on survival, suggesting that humidity may be more important for this species and life stage (Brown et al. 2023). We had total egg mortality at our lowest humidity treatments, 25% and 40%. No survival at 40% contrasts with some previous studies showing desiccation tolerance of *Ae. albopictus* eggs at 42% (e.g., Sota and Mogi 1992a, 1992b; Sota 1993). However, both of those experiments used substantially younger eggs (4 days old vs. our 4–6 week old eggs) and the comparably lower 25°C. As we have shown here, and demonstrated by Juliano et al. (2002), the temperature humidity interaction significantly influences survival outcomes. Our results therefore suggest that prolonged exposure to very low RH at common summer temperatures may be lethal even for a species with desiccation‐resistant eggs.

*Aedes albopictus* and other related vector species, such as *Aedes aegypti* and *Aedes triseriatus*, lay their eggs above the water line in small containers. Consequently, they will experience varying levels of microclimatic humidity as water levels fluctuate due to evaporation and rain in addition to daily changes in ambient humidity. If high temperatures are coupled with drought, the effect on egg survival will be more pronounced (Alto and Juliano 2001; Costanzo et al. 2005), although this may be limited in areas where people water their gardens (Little et al. 2017) or store water. Recent laboratory and large‐cage field trials have shown that adult mosquitoes behaviorally thermoregulate in response to temperature (Verhulst et al. 2020; Ziegler et al. 2023) by choosing cooler, more humid conditions (Kessler and Guerin 2008) and are more often found resting in cool, humid microclimates in the field (Sauer et al. 2021). Mosquitoes use hygrosensitive sensilla to detect humidity and water when looking for oviposition sites (Laursen et al. 2023), but it is unclear how or if *Aedes* mosquitoes use ambient temperature or humidity cues when choosing where to oviposit, which may have consequences for egg survival (Hilker et al. 2023). There is evidence that *Ae. aegypti* will delay oviposition in response to low humidity, however (Canyon et al. 1999). As studies of humidity preference in *Drosophila* have shown, desiccation and starvation status determine if a fly prefers high humidity locations in arenas, so there are likely complex interactions between female hydration status and oviposition choices that will be mediated by abiotic conditions (Giri et al. 2024). Future field studies should aim to determine if gravid females prefer cool and humid microclimates for oviposition and the potential consequences on egg survival.

Our next experiment aimed to test how eggs from different populations survive in more realistic field conditions. We looked at differences in egg survival from wild‐caught eggs from three local urban populations and eight of our lab strains outside in mid‐July, when *Ae. albopictus* abundance in Saint Louis, MO USA is high (Westby et al. 2021). We observed that wild eggs collected from two of our field sites had comparable survival rates to lab‐reared colony eggs when kept in ideal laboratory conditions. However, their survival rates dropped significantly during our field survival assay, indicating that wild mosquito eggs likely have low survivorship under natural conditions. Notably, two of our lab strains also exhibited very low survivorship in the field, suggesting that some populations are simply less capable of coping with stressful conditions. We regularly recorded temperatures above 32°C, which was our high temperature treatment in our lab experiment, but many of the populations had equivalent survival in the field as they did in our lab study. Relative humidity fluctuated between 50% and 100% during the 2 weeks of our field experiment. We saw from our laboratory experiment that no eggs survived in the 25% or 40% RH treatments and our field collected humidity data suggest that such low RH values are unlikely to be regularly experienced in shady field conditions in July. Interestingly, the Saint Louis, MO, population, where this study took place, had some of the lowest survival. Further research is needed to tease apart whether this is evidence of maladaptation or simply that this population has low egg survival in general. We suspect the latter, considering it also exhibited low survival in our first lab experiment.

We predicted that we would see low and varied survival from our wild eggs due to variability in resources available to females both in the larval habitat and as blood‐ and sugar‐feeding adults. For example, larval habitats can vary widely in the quality and quantity of nutrients available (Yee and Juliano 2006, 2012) which can carry over to affect the number of eggs a female produces (Costanzo et al. 2018) as can the vertebrate host source of the blood meal (de Swart et al. 2023; Yamany et al. 2026). Additionally, it is established that the size of a blood meal impacts the number of eggs produced, but our literature search did not reveal any papers that investigated the effects of larval or adult diet on egg survival in mosquitoes, although this has been documented in butterflies (Geister et al. 2008). We suspect that the differences in egg survival among the field‐collected populations were due to differences in maternal condition and plasticity in maternal provisioning (Gibbs and van Dyck 2009; Hilker et al. 2023) and not due to genetic differences. Further research using more populations collected across the urban matrix and tested in common gardens are needed to confirm this, but evidence of urban adaptation in the diapause trait in response to light pollution was not found in this species in the same metropolitan area (Westby and Medley 2020). Regardless, more research needs to be done to understand egg mortality in the field and the subsequent consequences for population growth, community interactions, and adaptation to the urban heat island.

Our final experiment tested for carry‐over effects of temperature experienced as eggs on subsequent larval survival during a simulated heatwave. We had very low larval survival overall with no statistically significant population differences, supporting our previous findings of minimal population divergence in thermal tolerance among larvae (Orlinick et al. 2024). There was a strong effect of egg temperature treatment on larval survival; almost no larvae were able to develop to the fourth instar when their eggs were held at the highest temperature treatment. This indicates that high temperatures experienced during the previous life stage have deleterious effects on larvae, rather than providing a positive priming effect and suggests that the abiotic conditions experienced as eggs have consequences for larval survival as well. Experimental evidence of heat stress experienced during the egg stage having negative effects on subsequent larvae has been documented in butterflies, moths, and damselflies (Potter et al. 2011; Janssens et al. 2017; Klockmann et al. 2017). However, most studies examining carry‐over effects of thermal stress on later life stages focus on larvae and adults so more experimental and observational evidence is needed to determine how consistent these findings are across different ectotherm taxa. Regardless, our results demonstrate that prolonged droughts and heat waves will have consequences for the population dynamics of *Ae. albopictus* in terms of both egg and larval survival. Because no larvae survived to adulthood and only a few pupated, our conclusions regarding carry‐over effects are limited to survival to the fourth instar under severe heatwave conditions. Thus, our experiment demonstrates that high egg temperatures strongly compromise larval survival under extreme thermal stress, but does not allow us to quantify effects on development time or adult traits.

In addition to local population dynamics, intraspecific variation in egg survival may have consequences for range expansions or contractions across species' ranges both due to shifting climate patterns (Angert et al. 2011), which vary by region (Melillo et al. 2014), and as a result of changes in the strength of interspecific competitive interactions (Legault et al. 2020; Åkesson et al. 2021). *Aedes albopictus* interacts strongly with other container breeding mosquitoes in the USA (Juliano and Lounibos 2005) such as *Ae. triseriatus* and *Ae. aegypti*. These species vary in their geographic range, upper thermal limits, and egg desiccation tolerance (Mogi et al. 1996; Juliano et al. 2002; Reinhold et al. 2018; Lahondère and Bonizzoni 2022). For example, *Ae. aegypti* has higher temperature limits and more desiccation resistant eggs compared to *Ae. albopictus* but is also an inferior resource competitor (Juliano and Lounibos 2005). Thus, in the southern USA where *Ae. aegypti* co‐occurs with *Ae. albopictus*, *Ae. aegypti* may gain an advantage as temperatures continue to rise and droughts become more frequent, increasing *Ae. albopictus* egg and larval mortality and relaxing competitive pressure in the larval stage (Costanzo et al. 2005; Lounibos et al. 2010).

While we were able to present the first published data on egg survival variation in different populations from North American *Ae. albopictus*, our study has several limitations that can be addressed in future work. First, although our common garden design reduces environmental variation, we cannot fully disentangle genetic from maternal effects, particularly for egg traits. Second, eggs were exposed to constant or simplified daily temperature and humidity regimes for relatively long durations, whereas microclimatic conditions in natural containers are more variable. Third, all colonies were maintained on a single artificial blood source, which may differentially affect maternal condition and egg production among populations. Fourth, we relied on calibrated visual estimates of egg viability rather than direct viability assays for every egg, introducing some uncertainty, especially under extreme treatments. Fifth, we only assessed survival at one time point which prevents us from estimating the rate that eggs died under different conditions or identifying the thresholds of exposure duration. Finally, our field experiment was conducted at a single site and over a single 2‐week period, so we did not capture interannual or regional variation. Future work combining quantitative genetic designs, genomic tools, and more natural field conditions will further refine our understanding about variation in *Aedes* egg survival across the diverse environmental conditions encountered throughout this species' exceptionally large global range.

## 5 Conclusions

In this paper, we have demonstrated that some populations of *Ae. albopictus* from across their USA range differ in egg desiccation tolerance in response to hot and dry conditions, with some populations consistently having low survival during times of thermal or hydric stress. Our common garden design suggests that egg desiccation has a heritable component and may evolve in response to climate change (Sota and Mogi 1992b; Sota 1993; Martín et al. 2025; Chakraborty et al. 2025) although our design cannot entirely rule out the role of maternal partitioning in egg traits that contribute to desiccation tolerance. We have shown that eggs that experience high temperatures produce larvae that are unable to develop to adulthood if high temperatures continue. Finally, we have argued that eggs are an understudied life stage, that egg mortality is likely highly variable in nature and important for population dynamics, and potentially also range expansions and contractions. These findings highlight the importance of egg survival and have implications for models that attempt to predict how this species will respond to climate change both in terms of disease transmission and range limits.

## Author Contributions

**Katie M. Westby:** conceptualization (lead), data curation (equal), formal analysis (lead), investigation (equal), methodology (equal), project administration (equal), supervision (equal), visualization (lead), writing – original draft (lead). **Laura Tayon:** conceptualization (equal), data curation (equal), investigation (equal), methodology (equal), project administration (equal), supervision (equal), writing – review and editing (equal). **Benjamin Orlinick:** investigation (equal), methodology (equal), writing – review and editing (equal). **Angela Smith:** conceptualization (equal), data curation (equal), investigation (equal), methodology (equal), project administration (equal), supervision (equal), writing – review and editing (equal). **Kim A. Medley:** formal analysis (equal), funding acquisition (equal), writing – review and editing (equal).

## Conflicts of Interest

The authors declare no conflicts of interest.

## Data Availability

The data, metadata, and SAS code have been uploaded to Figshare https://doi.org/10.6084/m9.figshare.30524186.
